# Supplementary material for: Properties and Functional Analysis of Two Chorismate Mutases from Maritime Pine
Source: Cells. 2024 May 28;13(11):929. doi: 10.3390/cells13110929 (PMC11171525; doi:10.3390/cells13110929)
Supplement: Supplementary file 1 [file cells-13-00929-s001.zip › Supplementary Materials/Supplementary Figure S1.pptx]

## Slide 1
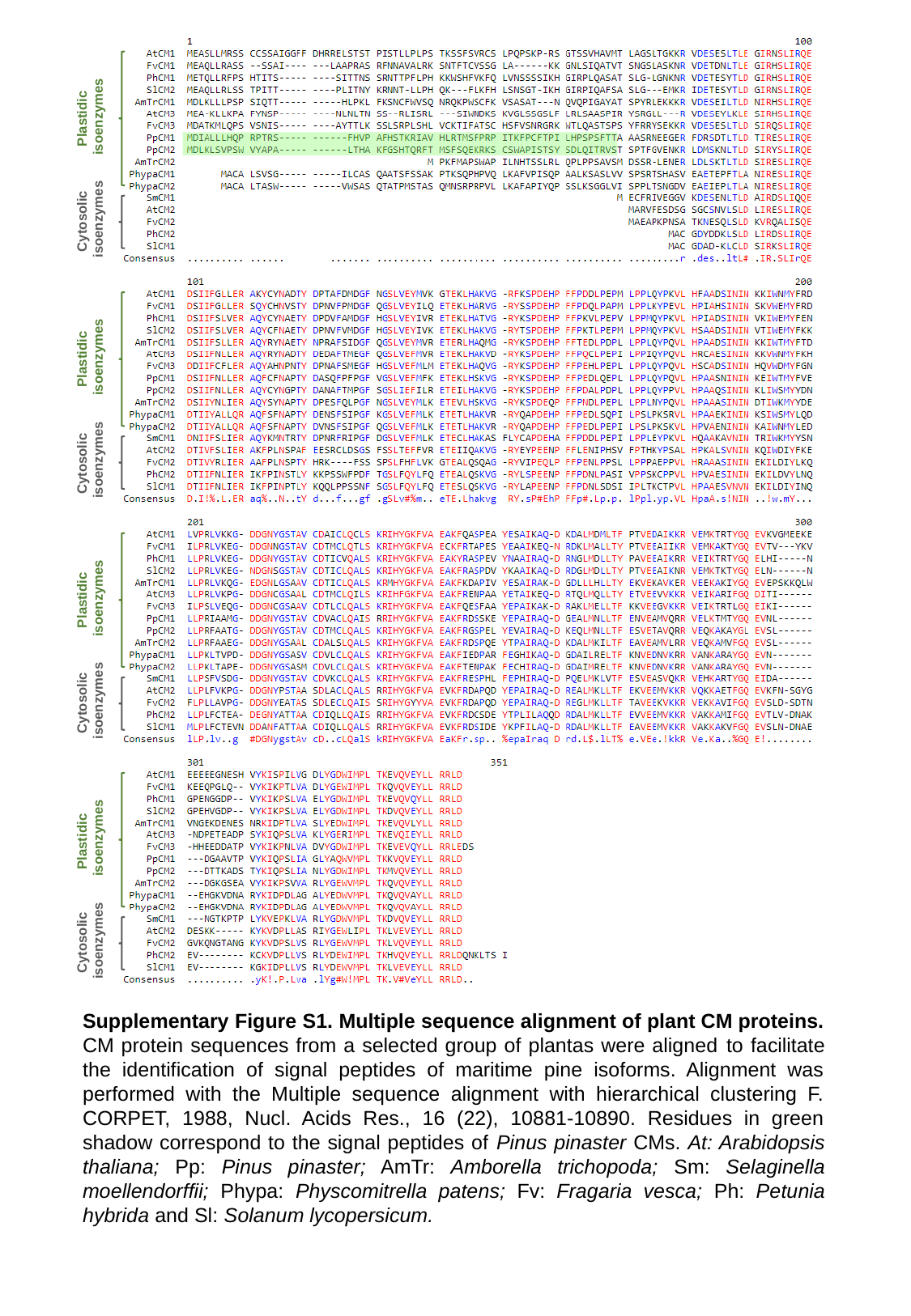

Plastidic
 isoenzymes
Cytosolic
 isoenzymes
Plastidic
 isoenzymes
Cytosolic
 isoenzymes
Plastidic
 isoenzymes
Cytosolic
 isoenzymes
Plastidic
 isoenzymes
Cytosolic
 isoenzymes
Supplementary Figure S1. Multiple sequence alignment of plant CM proteins. CM protein sequences from a selected group of plantas were aligned to facilitate the identification of signal peptides of maritime pine isoforms. Alignment was performed with the Multiple sequence alignment with hierarchical clustering F. CORPET, 1988, Nucl. Acids Res., 16 (22), 10881-10890. Residues in green shadow correspond to the signal peptides of Pinus pinaster CMs. At: Arabidopsis thaliana; Pp: Pinus pinaster; AmTr: Amborella trichopoda; Sm: Selaginella moellendorffii; Phypa: Physcomitrella patens; Fv: Fragaria vesca; Ph: Petunia hybrida and Sl: Solanum lycopersicum.
