# Supplementary material for: Properties and Functional Analysis of Two Chorismate Mutases from Maritime Pine
Source: Cells. 2024 May 28;13(11):929. doi: 10.3390/cells13110929 (PMC11171525; doi:10.3390/cells13110929)
Supplement: Supplementary file 1 [file cells-13-00929-s001.zip › Supplementary Materials/Supplementary Figure S2.pptx]

## Slide 1
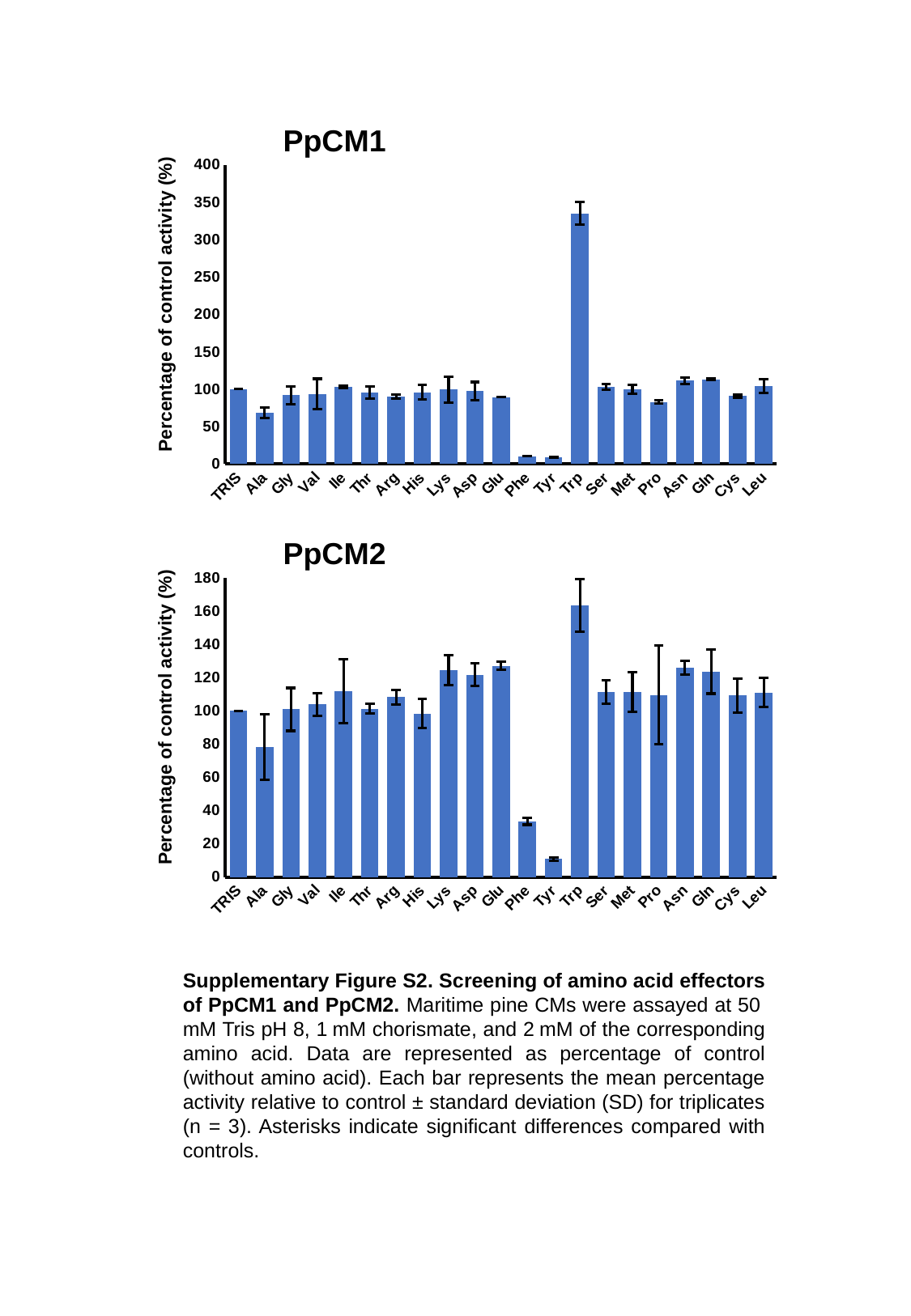

PpCM1
### Chart
| Category | |
|---|---|
| TRIS | 100.00357473036797 |
| Ala | 68.65086861104648 |
| Gly | 91.7956826951339 |
| Val | 93.53836004582462 |
| Ile | 103.11604959386534 |
| Thr | 95.50376963209021 |
| Arg | 90.09909640328145 |
| His | 95.986898401426 |
| Lys | 99.58885396602136 |
| Asp | 97.52561099342351 |
| Glu | 89.58912895112454 |
| Phe | 10.34 |
| Tyr | 8.754000000000001 |
| Trp | 334.72495000000004 |
| Ser | 103.24603105214291 |
| Met | 99.91317684397967 |
| Pro | 83.04737123813996 |
| Asn | 111.44384637811912 |
| Gln | 113.28948188931928 |
| Cys | 90.89650818833385 |
| Leu | 104.43190117566445 |Percentage of control activity (%)
PpCM2
### Chart
| Category | |
|---|---|
| TRIS | 100.0 |
| Ala | 78.24655385425245 |
| Gly | 100.79146870384515 |
| Val | 103.72799356414896 |
| Ile | 111.84683870265482 |
| Thr | 101.1759379060759 |
| Arg | 108.09080475440567 |
| His | 98.32748654624737 |
| Lys | 124.35327126475953 |
| Asp | 121.63738632578091 |
| Glu | 127.06049685555729 |
| Phe | 33.425889999999995 |
| Tyr | 10.74926 |
| Trp | 163.26217787847366 |
| Ser | 111.28004561791478 |
| Met | 111.32785900634835 |
| Pro | 109.52756047058917 |
| Asn | 125.85007473981634 |
| Gln | 123.60437168229149 |
| Cys | 109.16656319301254 |
| Leu | 110.94777241647085 |Percentage of control activity (%)
Supplementary Figure S2. Screening of amino acid effectors of PpCM1 and PpCM2. Maritime pine CMs were assayed at 50 mM Tris pH 8, 1 mM chorismate, and 2 mM of the corresponding amino acid. Data are represented as percentage of control (without amino acid). Each bar represents the mean percentage activity relative to control ± standard deviation (SD) for triplicates (n = 3). Asterisks indicate significant differences compared with controls.
